# The predictive value of CD4, CD8, and C—reactive protein in the prognosis of schistosomal and non-schistosomal colorectal cancer

**DOI:** 10.1186/s12876-023-02834-z

**Published:** 2023-06-05

**Authors:** Meihong Cheng, Hongyan Jing, Dacheng Bu, Jing Liu, Kui Lu, Jican Liu, Yanchao Xu, Ting Zhu, Yingyong Hou, Junxia Yao, Qilin Zhai, Weixia Wang

**Affiliations:** 1 Qingpu Branch of Zhongshan Hospital Affiliated to Fudan University, No. 1158 East Park Road, Qingpu District, Shanghai, 200032 P.R. China; 2 Department of Pathology, Zhongshan Hospital, Fudan University, Shanghai, 201700 P.R. China

**Keywords:** CD4 + T cell, CD8 + T cell, C-reactive protein, *Schistosomiasi*s, Colorectal cancer, Prognosis

## Abstract

**Background:**

Although *schistosomiasis* has been basically eliminated, it has not been completely extinction in China and occasional outbreaks occur in Europe in recent years. The relationship between inflammation caused by *Schistosoma* japonicum and colorectal cancer (CRC) is still obscure, and the inflammation based prognostic systems of schistosomal colorectal (SCRC) has rarely been reported.

**Aim:**

To explore the different roles of tumor infiltrating lymphocytes (TILs) and C-reactive protein (CRP) in SCRC and in Non-schistosomal CRC (NSCRC), providing a possible predictive system to evaluate outcomes and to improve the risk stratification for CRC patients, especially for CRC patients with *schistosomiasis*.

**Methods:**

Three hundred fifty-one CRC tumors were evaluated for density of CD4 + , CD8 + T cells and CRP in intratumoral and stromal compartments by immunohistochemical using tissue microarray.

**Results:**

There were no association between TILs and CRP and *schistosomiasis*. Multivariate analysis identified stromal CD4 (sCD4) (*p* = 0.038), intratumoral CD8 (iCD8) (*p* = 0.003), *schistosomiasis* (*p* = 0.045) as independent prognostic factors for overall survival (OS) in the whole cohort; and sCD4 (*p* = 0.006) and iCD8 (*p* = 0.020) were independent prognostic factors for OS in the NSCRC and SCRC set, respectively. Besides, we found that there were no differences of TILs and CRP, which were distributed in different areas of tumor tissue, between CRC patients with and without *schistosomiasis*.

**Conclusion:**

The results remind us that different subtypes of TILs have distinguished biological behavior and prognosis value in the immune microenvironment of NSCRC and SCRC patients. Meanwhile, the findings require us to stratify patients with *schistosomiasis* and this might facilitate patient counseling and management.

**Supplementary Information:**

The online version contains supplementary material available at 10.1186/s12876-023-02834-z.

## Introduction

*Schistosomiasis* is a chronic parasitic disease caused by a trematode blood fluke of genus *schistosoma* [1]. *Schistosomiasis* is a widespread endemic disease found in 74 countries [1] and over 250 million people are infected worldwide [2]. *Schistosomiasis* is now becoming a cause for concern in Europe, especially in southern Europe, because of climate change as well as infected travelers who return from endemic areas [3]. Qingpu District of Shanghai was once an endemic area of *schistosomiasis*, and the local people were deeply affected by it. There are still a large number of *schistosomiasis*-associated CRC patients left over from history. And *schistosomiasis*-associated CRC patients could be observed in our daily work occasionally.

It was suggested that *schistosomiasis* significantly correlated with increased colon cancer mortality in China [4]. Ming-Chai et al.`s study [5] revealed a similarity between chronic *schistosomiasis* and ulcerative colitis regarding predisposition to carcinoma in that pseudopolyposis, ectopic regenerating glands, epithelial proliferation and ulceration are common in both of them. They suggested a strong relation between *Schistosoma japonicum* and CRC. A similar conclusion was drawn by Yu et al. [6] from their studies on different types of schistosomal egg polyps. All these studies suggested that *shistosomiasis* is a risk factor for CRC. And our previous study suggested that *schistosomiasis* is an independent unbeneficial predictive factor for CRC [7]. However, some suggested that if there is an increase in the risk of CRC, it is small [6, 8]. Thus, the impacts of inflammation caused by *schistosomiasis* on CRC were necessary to be unraveled. However, related work has rarely been reported previously.

It was known that tumor-infiltrating lymphocytes (TILs) reflect an active inflammatory tumor microenvironment. And immunotherapeutic strategies harnessing the different components of the immune system to eliminate viable tumor cells are a promising therapeutic strategy. Several studies have been made to assess the prognostic significance of TILs in human cancers, and pronounced lymphocytic infiltration has been shown to be a prognostic parameter for better survival in CRC [9–11]. However, inflammation based prognostic systems for schistosomal CRC has never been reported in the literature. TILs are composed of various lymphocytes with diverse functions. CD4 + , CD8 + are the most common lymphocytes. Of them, CD8^+^ T cells play a crucial role in protective immunity against many infectious pathogens and can eradicate malignant cells by releasing perforins and granzymes, which may contribute to tumor cell death [12]. CD4 + T cells secreted immunoregulatory cytokines such as IFN-γand TNF that may induce cytolytic T cell responses in tumors. C-reactive protein (CRP), an acute phase reactant, primarily stimulates the innate immune system by facilitating phagocytosis, but also modulates adaptive immunity [13, 14]. Serum CRP has been shown to have prognostic value in CRC [15, 16]. However, the prognostic value of intratumor CRP remains unknown, especially in CRC patients with *schistosomiasis*.

With these considerations, we evaluated CD4 + , CD8 + and CRP in CRC by immunohistochemical and compared their different prognostic roles between CRC patients with and without *schistosomiasis*. These results may uncover the important role of CD4 + , CD8 + and CRP in schistosomal CRC and provide a possible predictive system to evaluate outcomes for patients with *schistosomiasis*.

## Materials and methods

### Patients and sample selection

In this retrospective study, 351 unselected CRC patients who received curative resection without preoperative chemotherapy at Qingpu Branch of Zhongshan Hospital affiliated to Fudan University, from January 2008 to August 2016 were included. Clinicopathological information of patients in this survey was collected from clinical records and pathology reports. The local Ethics Committee of Qingpu Branch of Zhongshan Hospital approved the protocol of this study, which was conducted in accordance with the Declaration of Helsinki. Written informed consent was obtained from all patients. The inclusion and exclusion criteria were as previously described [7].

### Tissue microarray construction

Tissue microarrays (TMA) were constructed as previously described [17]. Hematoxylin and eosin (H&E)-stained slides from tissue blocks had been reviewed for adequacy of the representative areas of interest with a high density of tumor cells. The corresponding regions were marked on archival formalin-fixed, paraffin-embedded (FFPE) tissue blocks. The representative areas (2 mm wide and 6 mm long) of the tumor were extracted and then vertically planted into the recipient block one by one according to the corresponding location. The planting surface was aggregated on the aggregation instrument. An array was constructed with a maximum of 40 cores.

### Immunohistochemical analysis

Three- to 5-μm thick CRC tissues were consecutively cut, subsequently dewaxed and rehydrated through graded alcohols. Slides were immunohistochemically stained in Roche Ventana Benchmark XT automated slide stainer (Ventana Medical Systems, Roche, France) according to the manufacturer`s instructions. Monoclonal and polyclonal anti-human antibodies were used for identification of CD4 + T cells (anti-CD4, 4B12, Dako), CD8 + T cells (anti-CD8, Ab4055, Abcam), CRP (Abcam, Cambridge, Massachusetts, Ab 32,412, rabbit monoclonal Y284).

#### Pathological assessment of CD4 and CD8 + T cell density and C-reactive protein

**Fig. 1:**
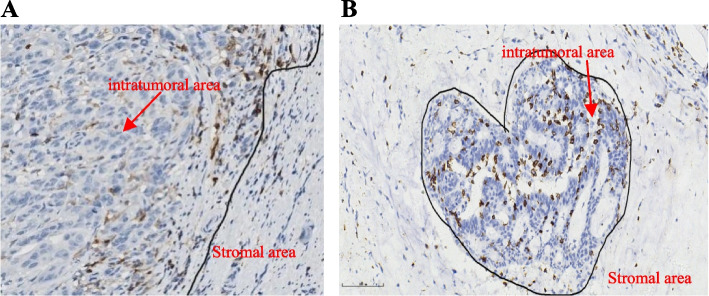
Immunohistochemical (IHC) staining of representative CD4 expression and CD8 expression positivity in different tumor areas. **A** CD4 expression positivity (× 100) in the intratumoral area (iCD4) (inside the circle) and stromal area (sCD4) (outside the circle). **B** CD8 expression positivity (× 200) in the intratumoral area (iCD8) (inside the circle) and stromal area (sCD8) (outside the circle)

The TMA slides were scanned using a scanner system (PRECICE 500B) at 40 × magnification. For CD4 and CD8, the densities of positively stained cells were evaluated on whole section slides using an image analysis system (Image J software, USA) (cells per square millimeter) (Fig. 1A, B). At least half of the core area was selected randomly, and the results of the calculated densities were extracted and put into an Excel file. Measurements were recorded as the mean number of positive cells per tissue unit in square millimetres as well as the number of positive cells among each 1-mm^2^ tissue units.

**Fig. 2:**
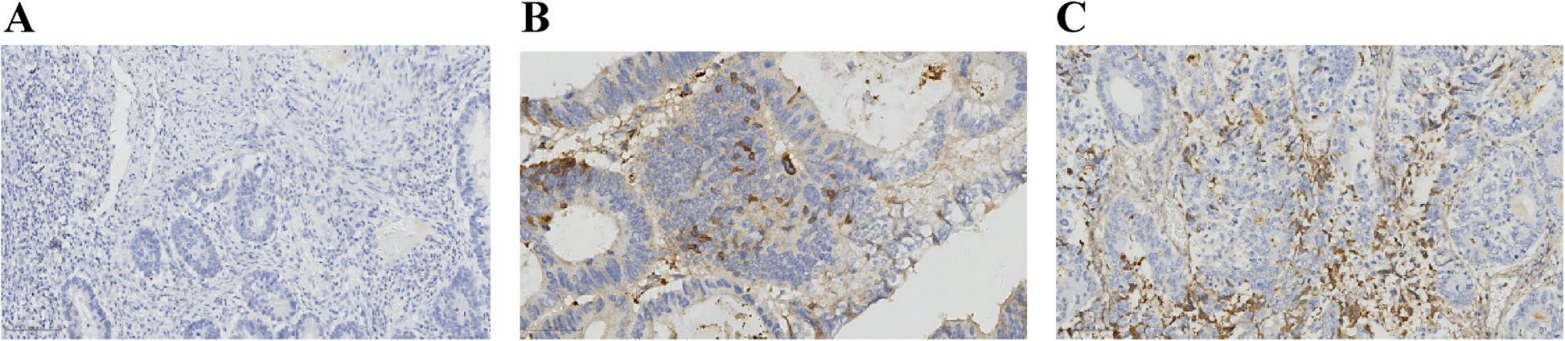
Immunohistochemical (IHC) staining pattern for C-reactive protein (CRP). **A** A negative immunohistochemical staining pattern (× 100); **B** Positive IHC staining pattern for tumoral CRP (× 200). **C** Positive IHC staining pattern for stromal CRP (× 100)

The C-reactive protein (CRP) staining were located in the stromal cells` and tumoral cells` cytoplasm in a diffused manner (Fig. 2A-C).

#### Detection of schistosome ova and assessment of tumor budding

Schistosome ova were observed in all of original HE stained formalin-fixed paraffin-embedded (FFPE) sections (usually 4–6 slides), which were examined at 10 × and 40 × magnification fields using a conventional light microscope by two pathologists who were blinded to the clinical data. The diagnosis of *schistosomiasis* was done by finding schistosome eggs in HE-stained slides (Sup. Fig. 1).

Tumor budding was defined as the presence of dedifferentiated single cells or small clusters of up to 5 cells at the invasive front of CRC [18]. The assessment of tumor budding was conducted as previously described [7]. Briefly, the 10-HPF method was used [19], the invasive front is first scanned at low magnification (4 × to 10 ×) to identify areas of highest budding density. Tumor buds are then counted under high magnification (40 ×), and the tumor budding count is reported. The evaluation of tumor budding was conducted by two pathologists who were blinded to the clinical data. Five tumor budding counts were used as breakthrough point. In brief, tumor bud counts greater than or equal to 5 were defined as the high group, otherwise as the low group.

### Statistical analysis

The associations between CD4 and CD8 and clinicopathologic features were analyzed using the chi-square (χ^2^) test. Time-to-event outcomes were defined from date of initially curative resection to date of last follow-up. Univariate analysis was based on the Cox proportional hazards regression model. A multivariate Cox forward stepwise regression model was used to detect independent predictors of survival. The survival curves were compared using Kaplan–Meier method and log-rank test. All tests were two sided, and a *P*-value of less than 0.05 was considered as statistical significance. Data were analyzed using SPSS version 22.0 software (SPSS Inc., Chicago, IL, USA).

## Results

### Study population

The median follow-up time was 62.4 (1.25–134.4) months. During the follow up, there was 41.6% (146 out of 351) patients died. Mean and median time to OS was 62.54 and 62.85, respectively. The clinicopathologic features of a total of 351 cases of CRC were summarized in Table 1. Briefly, the age of patients with *schistosomiasis* was dominantly older than that of patients without *schistosomiasis* (*p* < 0.001). Unexpectedly, there was no significant difference in morphology between CRC associated with *schistosomiasis* and that of without *schistosomiasis* (Table 1). In the whole cohort, the median age was 69 years (range 33–91), with frequencies between women 39% (137 out of 351) and men 61% (214 out of 351). The location of the tumor in 27% of patients was rectum, in left colon was 33% and in right colon was 40%. On the basis of the AJCC Staging Manual (seventh edition), 76% cases were histologically graded as well to moderately differentiated, and 24% were poorly differentiated. The most predominant histological type was adenocarcinoma (311 out of 351), mucinous and signet ring cell carcinoma were 11% (40 out of 351). Vessel and nerve involvement were identified in 122 (35%) and 31 (1%) tumors, respectively. Lymph node metastasis was identified in 144 (41%) patients. Stage I-II cases were accounted for 54% (190 out of 351), while stage III-IV cases were 46% (161 out of 351), respectively. *Schistosoma* eggs could be observed under microscope in almost 39% cases.

**Table 1:** Clinicopathological characteristics of the CRC cohort

Characteristics	All patients (*N* = 351)		Paitents with shistosomiasis (*N* = 137)		Patients without schistosomiasis (*N* = 214)	
	N	*%*	N	*%*	N	*%*
Age(> 60ys)	268	*76*	132	*96*	*136*	*64*
Gender(Male)	214	*61*	86	*63*	*126*	*59*
Tumor location						
Rectum	94	*27*	37	*27*	*57*	*27*
Left colon	115	*33*	51	*37*	*64*	*30*
Right colon	142	*40*	49	*36*	*93*	*43*
Tumor size(< 5 cm)	174	*50*	68	*50*	*106*	*50*
Tumor differentiation						
Well to moderately diff	267	*76*	102	*74*	*165*	*77*
Poor diff	84	*24*	35	*26*	*49*	*23*
Lymphovascular invasion (Negative)	122	*35*	90	*66*	*136*	*64*
Nervous invasion (Negative)	31	*1.0*	125	*91*	*194*	*91*
Tumor deposit	42	*1.2*	120	*88*	*189*	*88*
Colonic perforation (Yes)	13	*0.4*	5	*4*	*8*	*4*
Tummor budding (< 5 cells)	219	*62*	99	*72*	*156*	*73*
Ulceration (No)	149	*42*	77	*56*	*125*	*58*
Histological type						
Adenocarcinoma	311	*89*	119	*87*	*189*	*88*
Mucinous/SRCC	40	*11*	18	*13*	*25*	*12*
Pathological T stage						
T1-2	80	*23*	29	*21*	*51*	*24*
T3-4	271	*77*	108	*79*	*163*	*76*
Lymph node metastasis						
No	207	*59*	81	*59*	*126*	*59*
Yes	144	*41*	56	*41*	*88*	*41*
TNM stage						
I + II	190	*54*	76	*55*	*116*	*54*
III + IV	161	*46*	61	*45*	*98*	*46*
*schistosomiasis*	137	*39*	---	*---*	*---*	*---*

### Immunohistochemical findings

**Fig. 3:**
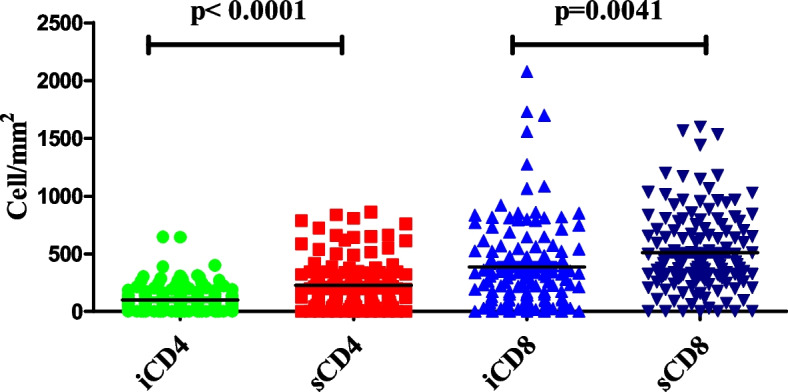
Heterogeneity of tumor-infiltrating immune cells. The density of iCD4 + , sCD4 + , iCD8 + , sCD8 + cells differed significantly according to tumor location (results were examined by using the unpaired *t*-test); Abbreviations: iCD4 = intratumoral CD4; sCD4 = stromal CD4; iCD8 = intratumoral CD8; sCD8 = stromal CD8

CD4 + and CD8 + cells were observed both in cancer stroma and within cancer epithelium (i.e., intraepithelial). Representative pictures of lymphocyte infiltration are shown in Fig. 1A, B. The distributions of CD4 + and CD8 + cell density in different areas are shown in Table 2 and Fig. 3. The optimum cutoff values of CD4 + T and CD8 + T cell density were determined by X-tile program, which were 29 cell/mm^2^ for intraepithelial CD4 (iCD4) (Table 2 and Sup Fig. 2A-C) and 145 cell/mm^2^ for stroma CD4 (sCD4) (Table 2 and Sup Fig. 3A-C) and 77 cell/mm^2^ for intraepithelial CD8 (iCD8) (Table 2 and Sup Fig. 4A-C) and 645 cell/mm^2^ for stroma CD8 (sCD8) (Table 2 and Sup Fig. 5A-C). Patients were divided into 2 groups for further analysis based on their respective cutoff values. The C-reactive protein (CRP) positive staining were located in the stromal cells` and tumoral cells` cytoplasm in a diffused manner (Fig. 2A-C). The CRP positive staining was defined as positive, whereas negative staining was defined as negative.

**Table 2:** Densities and cut-off values of the tumour infiltrating lymphocytes

	Density (cell/mm^2^)	Median value	Cutoff value	Low-density group(%)
iCD4	0–1857	*74*	*29*	31
sCD4	0–944	*203*	*145*	38
iCD8	0–2554	*291*	*77*	19
sCD8	0–2803	*462*	*645*	68

X-tile analysis of overall survival (OS) was performed using patients’ data collected from the pathological system of the Qingpu District Center for Disease Control and Prevention to determine the optimal cut-off value for iCD4, sCD4, iCD8, sCD8 density. The optimal cut-off value for iCD4 density was 29.0, for sCD4 was 145.0, for iCD8 was 77 and for sCD8 was 645 cell/mm^2^. Patients were divided into two groups: low-density group and high-density group based on the cutoff value of TILs

### Association of TILs density and CRP with clinicopathological features

The relationship between TILs density and patient demographics is listed in Table 3. No significant correlations were observed between iCD4 + T cell density and clinical characteristics, such as age, gender, TNM stage, tumor differentiation and so on (*p* > 0.05). There was also no correlation between sCD4 + T cell density and other clinical characteristics except for tumor budding (*p* = 0.031), lymph node metastasis (*p* = 0.045) and TNM stage (*p* = 0.001). Furthermore, there were significant association between iCD8 + T cell density and age (*p* < 0.001), tumor deposit (*p* = 0.032). In addition, there were significant association between sCD8 + T cell density and tumor deposit (*p* = 0.004). However, there were no significant association between TILs and shistosomiasis (*p* > 0.05).

**Table 3:** The association between clinicopathological characteristics and sTILs

Characteristic		iCD4		*P*	sCD4			*iCD8*		*P*	*sCD8*		*P*
		Low (*N* = 110)	High (*N* = 241)		Low (*N* = 135)	High (*N* = 216)	*P*	Low (*N* = 65)	High (*N* = 286)		Low (*N* = 238)	High (*N* = 113)	
Age(< 60ys)				0.223			0.520			< 0.001			0.500
	< 60	21	62		29	54		5	78		55	28	
	≥ 60	89	179		106	162		60	208		188	85	
Gender				0.198			0.574			0.095			0.813
	Male	72	140		79	133		45	167		148	68	
	Female	38	101		56	83		20	119		95	45	
Tumor site				0.676			0.773			0.442			0.506
	Rectum	29	65		34	60		16	78		66	30	
	Left colon	33	82		45	70		18	97		75	41	
	Right colon	48	94		56	86		31	111		102	42	
Tumor size				0.818			0.913			0.496			0.247
	< 5 cm	56	118		67	107		29	145		115	63	
	≥ 5 cm	54	123		68	108		36	141		128	50	
Tumor differentiation				0.998			0.699			0.337			0.499
	Well diff	84	183		100	167		47	220		182	88	
	Poor diff	25	58		35	49		18	66		61	25	
LVI				0.091			0.169			0.316			0.809
	Negative	63	163		80	146		39	187		158	70	
	Positive	47	78		55	70		26	99		85	43	0.550
Nervous invasion				0.693			0.706			0.998			
	Negative	99	220		124	195		59	260		219	104	
	Positive	11	21		11	21		6	26		24	9	
Tumor deposit				0.375			0.181			0.032			0.004
	≤ 2	94	215		115	194		52	257		206	106	
	> 2	16	26		20	22		13	29		37	7	
Colonic perforation				0.555			0.259			0.715			0.762
	No	105	233		128	210		62	276		233	110	
	Yes	5	8		7	6		3	10		10	3	
Ulceration				0.163			0.739			0.998			0.816
	No	57	145		80	122		37	165		141	65	
	Yes	53	96		55	94		28	121		102	48	
Pathological T stage				0891			0.432			0.871			0.168
	I + II	24	56		27	53		14	66		50	32	
	III	86	185		108	163		51	220		193	81	
Lymph node metastasis				0.198			0.045			0.677			0.481
	No	59	148		70	137		37	170		140	68	
	Yes	51	93		65	79		28	116		103	45	
TNM stage				0.160			0.001			0.275			0.416
	I + II	54	138		58	134		32	160		129	65	
	III + IV	56	103		77	82		33	126		114	48	
Tumor budding				0.636			0.031			0.888			0.635
	< 5 cells	64	146		73	137		39	171		139	72	
	≥ 5 cells	46	95		62	79		26	115		104	41	
Histological type				0.591			0.863			0.831			0.146
	Adenocarcinoma	94	214		117	191		57	251		208	104	
	Mucinous/SRCC	16	27		18	25		8	35		35	9	
Schistosomiasis				0.639			0.144			0.998			0.813
	Negtive	65	149		75	139		40	174		147	67	
	Positive	45	92		59	78		26	111		96	41	

----: Data is not applicable

*Abbreviations*: *N* Number, *LN* Lymph node, *iCD4* Intratumoral CD4, *sCD4* Stromal CD4, *iCD8* Intratumoral CD8, *sCD8* Stromal CD8, *LVI* Lymph vascular invasion, The association between schistosomiasis and clinicopathological characteristics was evaluated by using the Chi square and Fisher’s exact tests

The association between sCRP and tCRP and clinical characteristic were listed in Supplementary Table 1. Results demonstrated that sCRP was inversely associated with tumor size (*p* = 0.020) and colonic perforation (*p* = 0.001). Besides, tCRP was also negatively associated with colonic perforation (*p* = 0.001). Unexpectedly, there were no relationship between tCRP or sCRP and *schistosomiasis* as well as CD4 + T cells and CD8 + T cells.

### Univariate and multivariate regression analysis

In the whole cohort, univariate Cox regression analysis identified clinical factors significantly associated with OS (Table 4) were iCD4 (*p* = 0.015), sCD4 (*p* = 0.002), iCD8 (*p* < 0.001), age (*p* = 0.010), gender (*p* = 0.008), pathological T stage (*p* < 0.001), lymph node metastasis (*p* < 0.001), TNM stage (*p* < 0.001), tumor differentiation (*p* < 0.001), lymphovascular invasion (*p* < 0.001), tumor deposit (*p* < 0.001), tumor budding (*p* < 0.001) and *schistosomiasis* (*p* = 0.044), whereas only gender (*p* = 0.009), pathology T stage (*p* = 0.035), TNM stage (*p* < 0.001), sCD4 (*p* = 0.038), iCD8 (*p* = 0.003), *schistosomiasis* (*p* = 0.045) and tumor deposit (*p* < 0.001) were identified as independent risk factors for OS in multivariate regression analysis (Table 4).

**Table 4:** Univariate and multivariate Cox regression of clinicopathological for overall survival

Variable	All patients （*N*=351）		NSCRC （*N*=137）		SCRC （*N*=214）	
	*P*	HR(95%CI)	*P*	HR(95%CI)	*P*	HR(95%CI)
**Univariate analysis**						
iCD4	0.015	0.660(0.472-0.923)	0.076	0.662(0.419-1.044)	0.123	0.676(0.411-1.112)
sCD4	0.002	0.602(0.435-0.834)	<0.001	0.452(0.292-0.701)	0.75	0.924(0.566-1.507)
iCD8	<0.001	0.445(0.310-0.638)	0.002	0.459(0.283-0.745)	0.001	0.412(0.239-0.711)
sCD8	0.176	0.776(0.538-1.120)	0.057	0.612(0.369-1.015)	0.04	0.538(0.297-0.972)
Scrp	0.761	1.060(0.728-1.545)	0.478	1.231(0.685-2.210)	0.64	1.168(0.610-2.234)
tCRP	0.263	0.754(0.460-1.236)	0.796	0.906(0.430-1.908)	0.301	0.615(0.244-1.547)
Age (<60ys)	0.01	1.759(1.142-2.708)	0.122	1.454(0.905-2.336)	0.232	21.827(0.139-3436.270)
Gender (male/female)	0.008	1.602(1.129-2.271)	0.017	0.562(0.350-0.901)	0.307	1.311(0.779-2.207)
Tumor size(5cm)	0.913	1.018(0.728-1.400)	0.591	0.886(0.569-1.378)	0.32	1.282(0.786-2.089)
Tumor site						
Rectum		Refer		Refer		Refer
Left colon	0.908	1.025(0.676-1.553)	0.672	0.889(0.515-1.534)	0.484	1.263(0.657-2.427)
Right colon	0.464	0.859 (0.572-1.290)	0.054	0.590 (0.344-1.010)	0.13	1.631 (0.865-3.076)
Pathological T stage	<0.001	2.591(1.562-4.297)	0.001	3.363(1.620-6.980)	0.087	1.851(0.915-3.747)
Lymph node metastasis	<0.001	2.802(2.012-3.902)	<0.001	2.447(1.573-3.807)	<0.001	3.552(2.141-5.894)
TNM stage	<0.001	3.197(2.271-4.501)	<0.001	2.764(1.752-4.358)	<0.001	4.219(2.497-7.128)
Tumor differentiation	<0.001	1.889(1.334-2.674)	0.003	2.009(1.259-3.206)	0.054	1.668(0.991-2.809)
LVI	<0.001	3.251(1.987-5.318)	<0.001	2.816 (1.808-4385)	0.275	1.321 (0.801-2.180)
Nervous invasion	0.14	1.497(0.876-2.559)	0.391	1.424 (0.710-2.857)	0. 206	1.727 (0.741-4.024)
Tumor deposit	<0.001	4.006(2.686-5.973)	<0.001	3.973(2.359-6.692)	<0.001	4.138(2.205-7.769)
Colonic perforation	0.541	0.700(0.223-2.198)	0.763	1.194(0.377-3.786)	0.5	0.506(0.070-3.657)
Tummor budding	<0.001	2.028(1.400-2.938)	<0.001	2.824(1.813-4.400)	0.237	1.354(0.819-2.238)
*Schistosomiasis*	0.044	1.399(1.009-1.940)	—	—	—	—
Ulceration	0.624	0.9205(0.660-1.282)	0.744	1.077(0.691-1.676)	0.971	1.008(0.670-1.514)
Histological type	0.921	1.025(0.626-1.680)	0.283	1.400 (0.758-2.586)	0.467	0.760(0.362-1.594)
**Multivariate analysis**						
Age	—	—	—	—	0.967	1669993.854(0.000-4.660E+255)
Gender	0.009	1.614(1.127-2.310)	0.023	1.771(1.084-2.896)	—	—
Pathological T stage	0.035	1.756(1.041-2.962)	0.046	2.182(1.015-4.688)	—	—
TNM stage	< 0.001	2.225(1.512-3.273)	—	—	—	—
Lymph node metastasis	—	—	—	—	<0.001	2.715(1.570-4.696)
Tumor differentiation	—	—	—	—	0.254	1.366(0.800-2.331)
sCD4	0.038	0.698(0.497-0.980)	0.006	0.527(0.334-0.859)		
iCD8	0.003	0.565(0.388-0.822)	—	—	0.02	0.518(0.298-0.903)
sCD8	—	—	—	—	0.156	0.648(0.356-1.180)
Tumor Budding	—	—	0.034	1.675(1.039-2.700)	—	—
*Schistosomiasis*	0.045	1.404(1.007-1.958)	—	—	—	—
Lymphovascular invasion	—	—	0.061	1.590(0.978-2.586)	—	—
Tumor deposit	<0.001	2.233(1.430-3.488)	0.002	2.488(1.413-4.380)	0.015	2.257(1.158-4. 400)

—: Data is non-significant

*Abbreviation*: *NSCRC* Non-schistosomal CRC, *SCRC* Schistosomal CRC, *CI* Confidence interval, *HR* Hazard ratio, *LN* Lymph node, *iCD4* Intratumoral CD4, *sCD4* Stromal CD4, *iCD8* Intratumoral CD8, *sCD8* Stromal CD8, *sCRP* Stromal C-reactive protein, *tCRP* Tumoral C-reactive protein, *LVI* Lymph vascular invasion. *p* < 0.05 was defined as the criterion for variable deletion when performing backward stepwise selection

In the NSCRC set, univariate Cox regression analysis identified clinical factors significantly associated with OS (Table 4) were sCD4 (*p* < 0.001), iCD8 (*p* = 0.002), gender (*p* = 0.017), pathological T stage (*p* = 0.001), lymph node metastasis (*p* < 0.001), TNM stage (*p* < 0.001), tumor differentiation (*p* = 0.003), lymphovascular invasion (*p* < 0.001), tumor deposit (*p* < 0.001) and tumor budding (*p* < 0.001). Variables demonstrating a significant effect on OS were included in the multivariate analysis. Gender (*p* = 0.023), pathology T stage (*p* = 0.046), sCD4 (*p* = 0.006), tumor budding (*p* = 0.034), and tumor deposit (*p* < 0.0001) were identified as independent prognostic factors that associated with OS in the set in multivariate regression analysis (Table 4).

In SCRC set, univariate Cox regression analysis identified clinical factors statistically significantly associated with OS (Table 4) were iCD8 (*p* = 0.001), sCD8 (*p* = 0.040), lymph node metastasis (*p* < 0.001), TNM stage (*p* < 0.001) and tumor deposit (*p* < 0.001), whereas only lymph node metastasis (*p* < 0.001), iCD8 (*p* = 0.020) and tumor deposit (*p* < 0.001) were identified as independent prognostic factors that associated with OS in this set in multivariate regression analysis (Table 4). Unexpectedly, there were no association between stromal CRP and tumoral CRP and OS in the whole cohort and subgroups.

### Kaplan–Meier analysis of OS

**Fig. 4:**
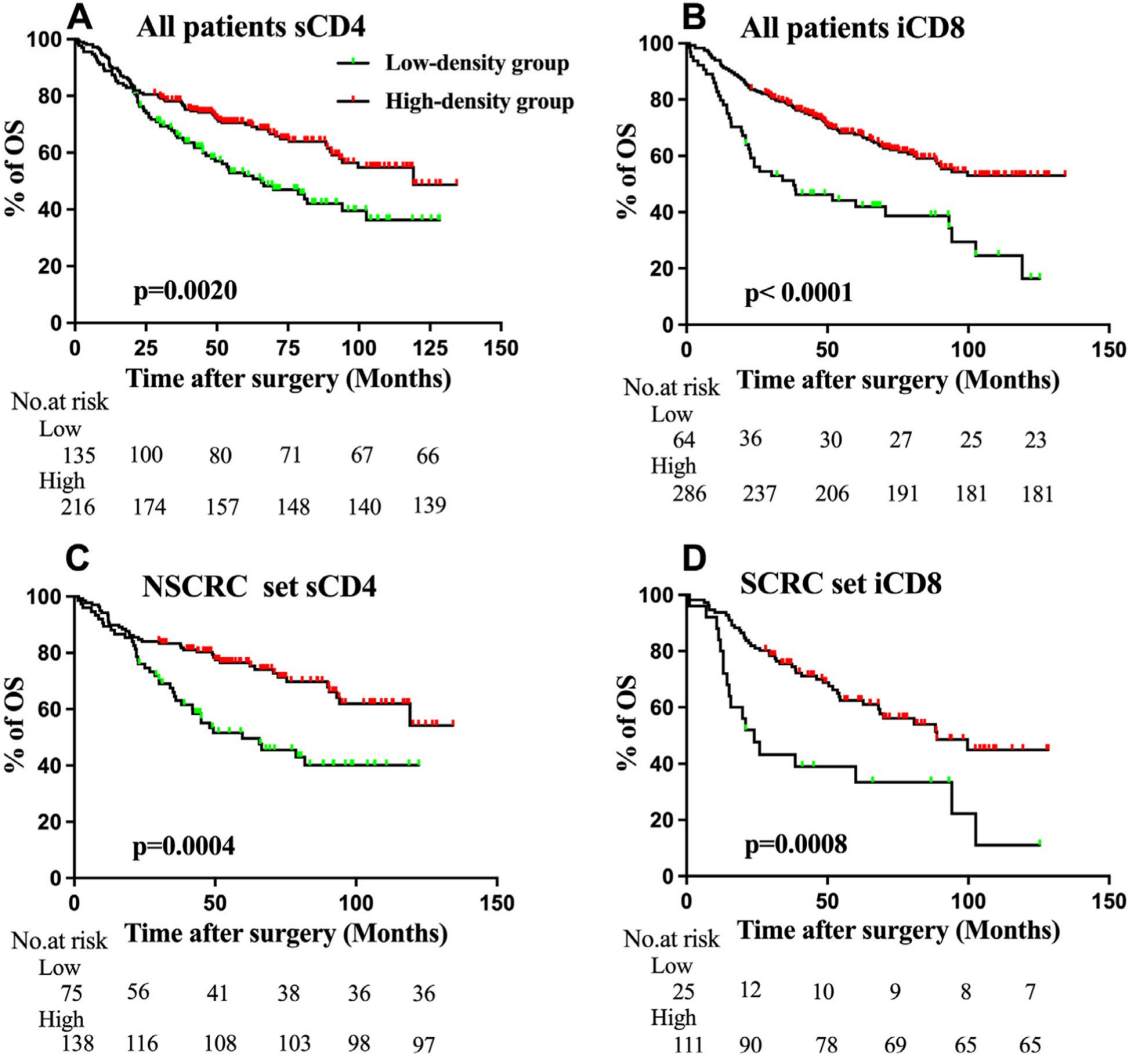
Kaplan–Meier (K-M) curves for overall survival (OS) of colorectal cancer (CRC) patients with low or high TILs density. **A**.OS analysis of stromal CD4 + T cell density in the whole cohort; **B**. OS analysis of intratumoral CD8 + T cell density in the whole cohort; **C**. OS analysis of stromal CD4 + T cell density in schistosomal CRC (NSCRC) patients; **D**. OS analysis of intratumoral CD8 + T cell density in non-schistosomal CRC (SCRC) patients

Kaplan–Meier analysis, which was based on the cutoff value of the density of CD4 + T cells and CD8 + T cells in different tumor areas, was conducted to assess the variables in OS among different groups. In the whole cohort, compared to the low-density group, patients in the sCD4 and iCD8 high-density group experienced significantly higher OS (*p* = 0.0020, *p* < 0.001; Fig. 4A and B). In the NSCRC set, patients in the sCD4 high-density group gained beneficial OS compared to patients in the low-density group (*p* = 0.0004, Fig. 4C). Furthermore, patients in the iCD8 high-density group also possessed favorable survival compared to that of in the low-density group (*p* = 0.0008, Fig. 4D). There was no association between sCRP or tCRP and OS in any subgroups (data was not shown).

### Comparison of TILs density and positive stained CRP in CRC patients with and without *schistosomiasis*

We next compared the density distribution of CD4 + and CD8 + T cells in CRC patients with and without *schistosomiasis* (data was not shown). The distribution of CD4 + T cells in stromal or tumoral area between NSCRC and SCRC groups had no significant differences. Similarly, the distribution of CD8 + T cells in stromal or tumoral area also had no significant differences between the NSCRC and SCRC group. The expression positivity rate of stromal CRP was 22% and 30% in the NSCRC group and SCRC group, respectively. Besides, the expression positivity rate of tumoral CRP was 14% and 15% in the NSCRC group and SCRC group, respectively.

## Discussion

*Schistosomiasis* leads to inflammatory changes in CRC patients is well studied. However, direct evidences supporting inflammatory changes caused by inflammatory response after *schistosomiasis* infection are still lacking. Moreover, inflammation based prognostic systems for CRC were pronounced, but related predictors for schistosomal CRC have never been reported previously. In the present study, we found that CD4 + and CD8 + T cells distributed in different tumor areas were correlated with less aggressive tumor characteristics, but CRP distributed in different tumor areas were associated with more aggressive tumor features. In addition, results suggested that iCD4, sCD8, *schistosomiasis*, gender, pathological T stage, TNM stage and tumor deposit were independent prognostic factors for OS in the whole cohort; In the NSCRC set, sCD4, tumor budding, gender, pathological T stage and tumor deposit were independent prognostic factors for OS; In the SCRC set, iCD8, lymph node metastasis and tumor deposit were independent prognostic factors for OS. Furthermore, we found that there were no differences of TILs densities and CRP expression positivity, which were distributed in different areas of tumor tissue, between CRC patients with and without *schistosomiasis*.

Our results demonstrated that iCD4 and sCD8 were associated with favorable OS for CRC patients. However, when patients were divided into NSCRC and SCRC set based on status of schistosomal infection, sCD4 and iCD8 were independent prognostic factor for NSCRC patients and for SCRC patients, respectively. These results remind us that TILs distributed in different tumor areas have distinguished biological behavior and function, and the immune microenviroment is different in NSCRC and SCRC patients. Meanwhile, the findings require us to stratify patients with *schistosomiasis* and might facilitate patient counseling and management. The findings also pave the way for customized chemotherapy in CRC patients with *schistosomiasis* and without *schistosomiasis* individually based on the infiltration of TILs, because CRC patients with high levels of TILs could have improved outcome with 5-fluorouracil-based chemotherapy.

CD8 + T cells are cytotoxic T lymphocytes that directly attack cancer cells and play a central role in anti-cancer immunity [20]. Previous studies revealed substantial evidence that the density of CD8 + TILs was associated with the favorable survival in patients with various types of cancer [20–22]. Consistent with previous reports, this study confirmed the usefulness of CD8 + T-cell densities as prognostic factors (Table 4 and Fig. 4). CD4 + T cell density has been reported to be a unbeneficial prognostic factor in other types of cancers, e.g. lung, renal, prostate and breast cancer [23–26]. On the contrary, Taichi Kuwahara et al.`s study showed that CD4 + T cell densities were associated with favorable clinical outcomes [27]. This is consistent with our results. The reason for this discrepancy remains unclear, but it may be because the function of CD4 + T cells within the tumor microenvironment—i.e. in immune response activation or immunosuppression—may differ depending on the cancer type. Besides, we found that CRP was not associated with clinical outcome. This may be because CRP is a good predictor for clinical outcomes in the acute phase [15, 28, 29], or may be serum CRP is more suitable as a prognostic predictor. It was well known that *schistosoma* egg deposited in the intestine provoke immune response and lead to chronic inflammation, and then lead to *shisotomiasis*. It was speculated that the densities of TILs in NSCRC and SCRC patients were different. However, our results showed that the densities of TILs and CRP expression levels in the two sets were similar. It may be because patients at diagnosis were not in the acute stage, but in the chronic stage. In addition, this may be explained that patients with *schistosomiasis* are obviously older than patients without *schistosomiasis* (Table 1), and the immune response of the elderly is weaker than that of the young.

Our retrospective study had several limitations. First, we do recognized the limitation of utilizing a TMA approach to assess expression of a biomarker that may only be locally present in samples, raising the possibility of false negatives, which could possibly change the significance of TILs in CRC. Second, because all results were generated at a single institution, the uniformity of the results may be low. Further work will be needed to validate the present results in a larger cohort of multicenters. Besides, validation studies may be performed on whole tissue slides to optimize the selection of regions which is most suitable for TILs assessment. Thirdly, the diagnosis of *schistosomiasis* was done by finding schistosome eggs in HE-stained slides. This will lead to positive cases miss and to generate data bias. We tried to use other methods to detect *schistosomiasis* related indicators, such as imaging data and serological tests. However, imaging data was not completed and serum could not be collected as the specimen used in this study was years ago. At present, more comprehensive materials related to *schistosomiasis* were collecting and will be used to validate results from this study.

## Conclusion

In present study, iCD4 + and sCD4 + T cell densities were independent beneficial prognostic factors for NSCRC patients and SCRC patients, respectively. We believe that this study is the first to report the prognostic significance of TILs and CRP in *schistosomiasis*-associated CRC patients. Hence, the results in this study remind us that different subtypes of TILs, which distributed in different tumor areas, have distinguished biological behavior and prognosis value in the immune microenviroment of NSCRC and SCRC patients. Meanwhile, the findings require us to stratify patients with *schistosomiasis* and might facilitate patient counseling and management.

## Supplementary Information

**Additional file 1: Supplementary Table 1.** The association between clinicopathological characteristics and tumoral and stromal C-reactive protein. **Sup Fig. 1.** Typical sample of schistosomiasis-associated colorectal cancer, the red arrows indicate schistosome ova. **Sup Fig. 2.** Determination of cut-off values of intratumoral CD4 density of TMAs and survival analyses. **Sup Fig. 3.** Determination of cut-off values of stromal CD4 density of TMAs and survival analyses. **Sup Fig. 4.** Determination of cut-off values of intratumoral CD8 density of TMAs and survival analyses. **Sup Fig. 5.** Determination of cut-off values of stromal CD8 density of TMAs and survival analyses.

## Data Availability

The datasets used and/or analyzed during the current study are available from the corresponding authors on reasonable request.

## Abbreviations

CRC: Colorectal cancer
SCRC: Schistosomal colorectal cancer
NSCRC: Non-Schistosomal colorectal cancer
TILs: Tumor infiltrating lymphocytes
CRP: C-reactive protein
TMA: Tissue microarray
iCD4: Intratumoral CD4
sCD4: Stromal CD4
iCD8: Intratumoral CD8
sCD8: Stromal CD8
OS: Overall survival
H&E: Hematoxylin and eosin
FFPE: Formalin fixed paraffin-embedded
