# Supplementary material for: The predictive value of CD4, CD8, and C—reactive protein in the prognosis of schistosomal and non-schistosomal colorectal cancer
Source: BMC Gastroenterol. 2023 Jun 5;23:194. doi: 10.1186/s12876-023-02834-z (PMC10240683; doi:10.1186/s12876-023-02834-z)
Supplement: Supplementary file 1 — Additional file 1: Supplementary Table 1. The association between clinicopathological characteristics and tumoral and stromal C-reactive protein. Sup Fig. 1. Typical sample of schistosomiasis-associated colorectal cancer, the red arrows indicate schistosome ova. Sup Fig. 2. Determination of cut-off values of intratumoral CD4 density of TMAs and survival analyses. Sup Fig. 3. Determination of cut-off values of stromal CD4 density of TMAs and survival analyses. Sup Fig. 4. Determination of cut-off values of intratumoral CD8 density of TMAs and survival analyses. Sup Fig. 5. Determination of cut-off values of stromal CD8 density of TMAs and survival analyses. [file 12876_2023_2834_MOESM1_ESM.doc]

| **Supplementary Table 1**. The association between clinicopathological characteristics and tumoral and stromal C-reactive protein (CRP) | | | | | | | |
| --- | --- | --- | --- | --- | --- | --- | --- |
| Characteristic |  | tCRP | | *P value* | sCRP | |  |
|  |  | Negative  (N=299) | Positive  (N=52) |  | Negative  (N=263) | Positive  (N=88) | *P value* |
| Age(＜60ys) |  |  |  | 0.291 |  |  | 0.111 |
|  | ＜60 | 74 | 9 |  | 68 | 15 |  |
|  | ≥60 | 225 | 43 |  | 195 | 73 |  |
| Gender |  |  |  | 0.998 |  |  | 0.533 |
|  | Male | 181 | 21 |  | 104 | 35 |  |
|  | Female | 118 | 31 |  | 159 | 53 |  |
| Tumor site |  |  |  | 0.119 |  |  | 0.972 |
|  | Rectum | 86 | 8 |  | 78 | 16 |  |
|  | Left colon | 94 | 21 |  | 83 | 32 |  |
|  | Right colon | 119 | 23 |  | 102 | 40 |  |
| Tumor size |  |  |  | 0.177 |  |  | 0.020 |
|  | ＜5cm | 153 | 21 |  | 140 | 34 |  |
|  | ≥5cm | 146 | 31 |  | 123 | 54 |  |
| Differentiation |  |  |  | 0.482 |  |  | 0.885 |
|  | Well diff. | 225 | 42 |  | 199 | 68 |  |
|  | Poor diff. | 74 | 10 |  | 64 | 20 |  |
| Lymphovascular invasion |  |  |  | 0.876 |  |  | 0.519 |
|  | Negative | 193 | 33 |  | 169 | 57 |  |
|  | Positive | 106 | 19 |  | 94 | 31 |  |
| Nervous invasion |  |  |  | 0.196 |  |  | 0.283 |
|  | Negative | 269 | 50 |  | 237 | 82 |  |
|  | Positive | 30 | 2 |  | 26 | 5 |  |
| LNs positive for CRC |  |  |  | 0.486 |  |  | 0.127 |
|  | ≤2 | 265 | 44 |  | 236 | 73 |  |
|  | ＞2 | 34 | 8 |  | 27 | 15 |  |
| Colonic perforation |  |  |  | 0.001 |  |  | 0.001 |
|  | No | 293 | 45 |  | 259 | 79 |  |
|  | Yes | 6 | 7 |  | 4 | 9 |  |
| Ulceration |  |  |  | 0.131 |  |  | 0.319 |
|  | No | 167 | 35 |  | 147 | 55 |  |
|  | Yes | 132 | 17 |  | 116 | 33 |  |
| Pathological T stage |  |  |  | 0.593 |  |  | 0.463 |
|  | I+ II | 70 | 10 |  | 63 | 17 |  |
|  | III | 229 | 42 |  | 200 | 71 |  |
| Lymph node metastasis |  |  |  | 0.648 |  |  | 0.381 |
|  | No | 178 | 29 |  | 159 | 48 |  |
|  | Yes | 121 | 23 |  | 104 | 40 |  |
| TNM stage |  |  |  | 0.763 |  |  | 0.324 |
|  | I+II | 165 | 27 |  | 148 | 44 |  |
|  | III+ IV | 134 | 25 |  | 115 | 44 |  |
| Tumor budding |  |  |  | 0.504 |  |  | 0.170 |
|  | ＜5 cells | 215 | 40 |  | 186 | 69 |  |
|  | ≥5 cells | 84 | 12 |  | 77 | 19 |  |
| Histological type |  |  |  | 1.000 |  |  | 0.853 |
|  | Adenocarcinoma | 262 | 4 |  | 230 | 78 |  |
|  | Mucinous/SRCC | 37 | 6 |  | 33 | 10 |  |
| iCD4 |  |  |  | 0.628 |  |  | 0.426 |
|  | Low-density | 92 | 18 |  | 79 | 31 |  |
|  | High-density | 207 | 34 |  | 184 | 57 |  |
| sCD4 |  |  |  | 0.558 |  |  | 0.614 |
|  | Low-density | 115 | 20 |  | 99 | 36 |  |
|  | High-density | 184 | 32 |  | 164 | 52 |  |
| iCD8 |  |  |  | 0.848 |  |  | 0.428 |
|  | Low-density | 55 | 10 |  | 46 | 19 |  |
|  | High-density | 244 | 42 |  | 217 | 69 |  |
| sCD8 |  |  |  | 0.873 |  |  | 0.511 |
|  | Low-density | 202 | 36 |  | 181 | 57 |  |
|  | High-density | 97 | 16 |  | 82 | 31 |  |
| Schistosomiasis |  |  |  | 0.878 |  |  | 0.102 |
|  | Negative | 183 | 31 |  | 167 | 47 |  |
|  | Positive | 116 | 21 |  | 96 | 41 |  |
| ----:Data is not applicable; Abbreviation:N =Number; LN= Lymph node. The association between schistosomiasis and clinicopathological characteristics was evaluated by using the  Chi square and Fisher’s exact tests. | | | | | | | |


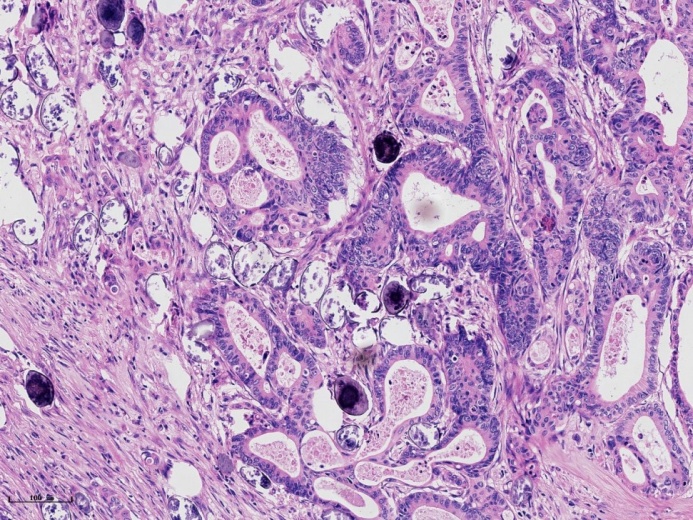


**Sup Fig.1.** Typical sample of schistosomiasis-associated colorectal cancer, the red arrows indicate schistosome ova (HE, ×100)

A B C


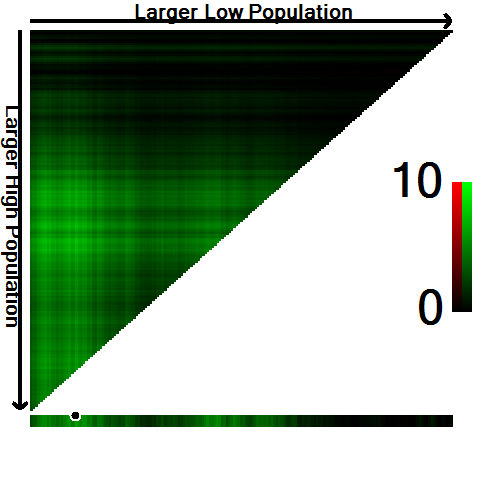

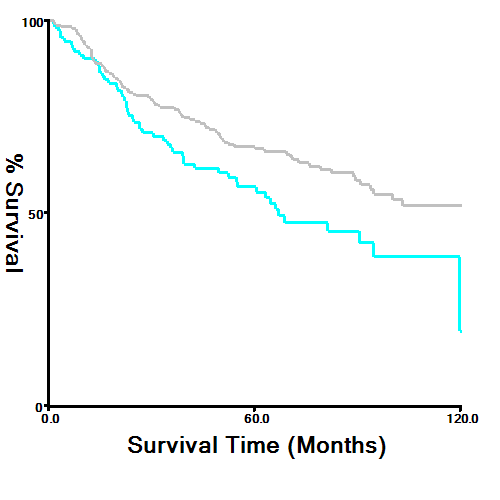

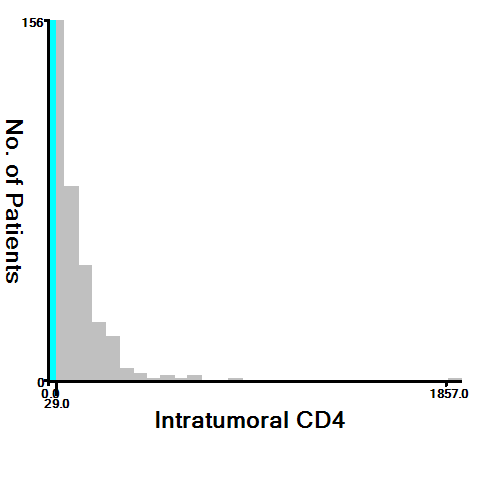


black circle

**Sup Fig. 2.** Determination of cut-off values of intratumoral CD4 (iCD4) density of TMAs and survival analyses. X-tile analysis of overall survival (OS) was performed using patients’ data collected from the pathological system of the Qingpu District Center for Disease Control and Prevention to determine the optimal cut-off value for iCD4 density. The optimal cut-off values highlighted by the black circles in left panels are shown in histograms of the entire cohort (middle panels), and Kaplan-Meier plots are displayed in right panels. P values were determined by using the cut-off values defined in the whole cohort. The optimal cut-off value for iCD4 density was 29.0 (p＜0.0001).

A B C


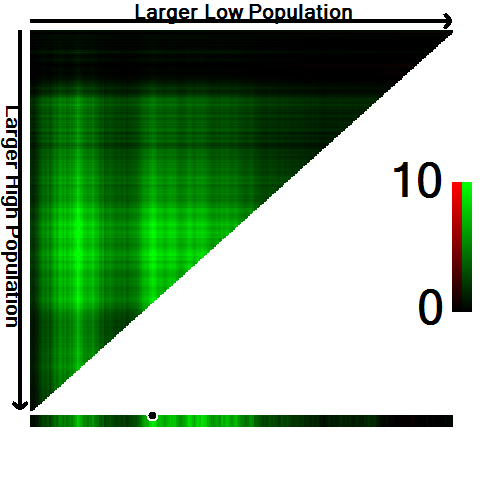

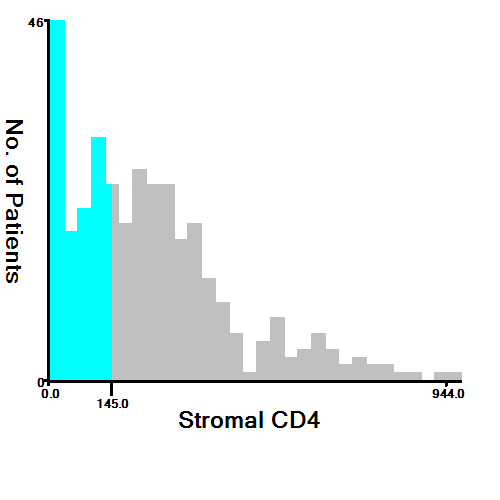

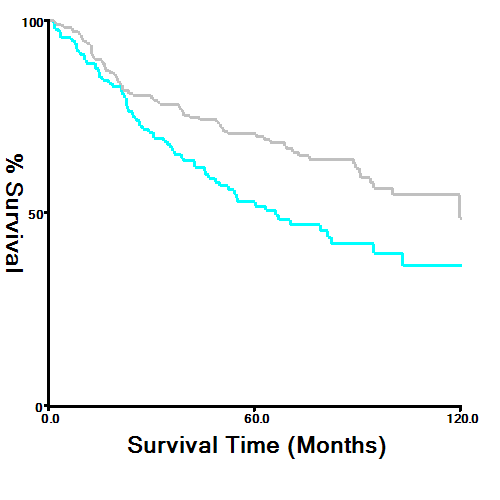


black circle

**Sup Fig. 3.** Determination of cut-off values of stromal CD4 (sCD4) density of TMAs and survival analyses. The optimal cut-off values highlighted by the black circles in left panels are shown in histograms of the entire cohort (middle panels), and Kaplan-Meier plots are displayed in right panels. P values were determined by using the cut-off values defined in the whole cohort. The optimal cut-off value for sCD4 density was 145.0 (p=0.02133).

A B C


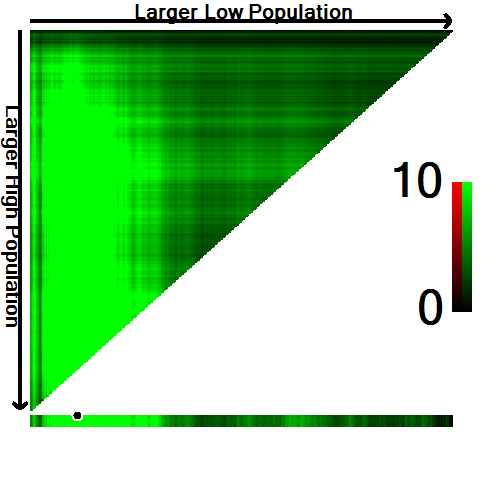

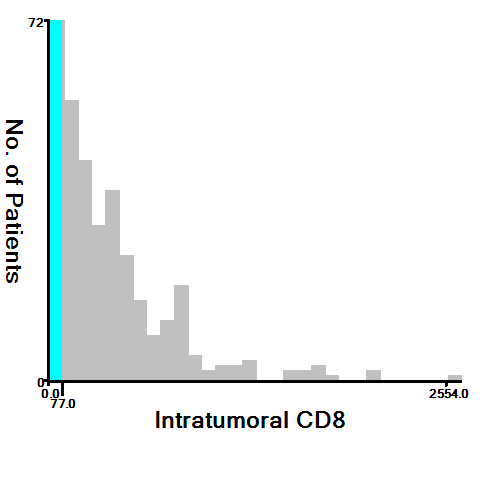

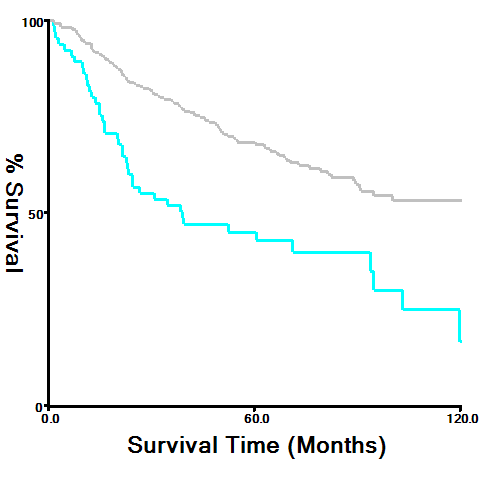


black circle

**Sup Fig. 4.** Determination of cut-off values of intratumoral CD8 (iCD8) density of TMAs and survival analyses. The optimal cut-off values highlighted by the black circles in left panels are shown in histograms of the entire cohort (middle panels), and Kaplan-Meier plots are displayed in right panels. P values were determined by using the cut-off values defined in the whole cohort. The optimal cut-off value for iCD8 density was 77.0 (p=0.0005).

A B C


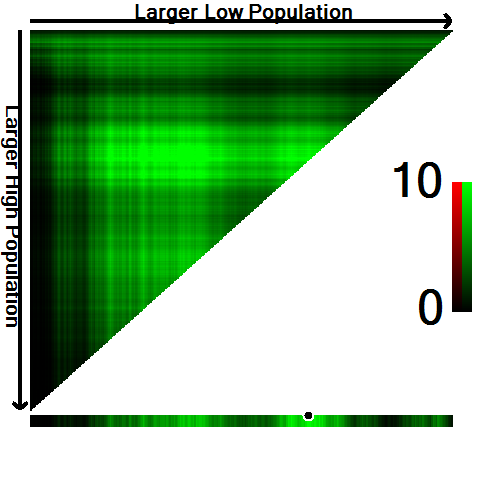

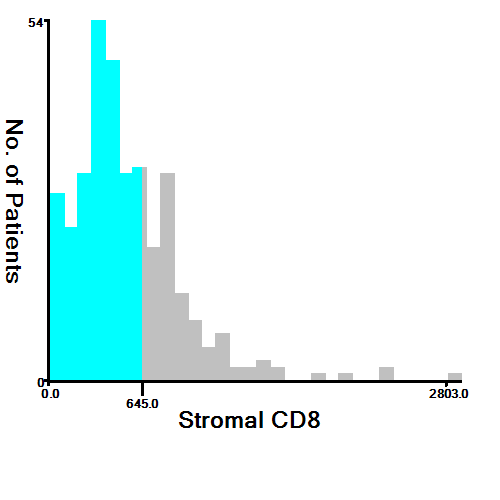

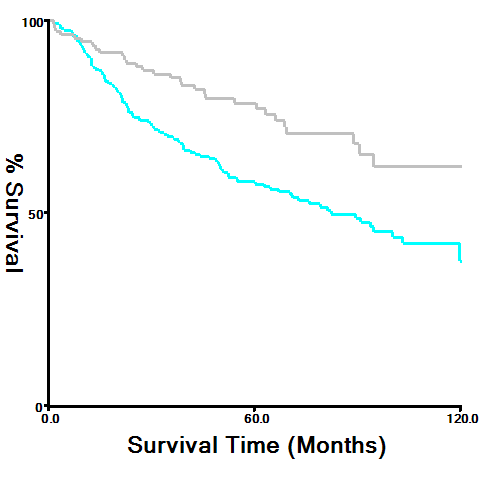


black circle

**Sup Fig. 5.** Determination of cut-off values of stromal CD8 (sCD8) density of TMAs and survival analyses. The optimal cut-off values highlighted by the black circles in left panels are shown in histograms of the entire cohort (middle panels), and Kaplan-Meier plots are displayed in right panels. P values were determined by using the cut-off values defined in the whole cohort. The optimal cut-off value for sCD8 density was 645.0 (p=0.0246).
